# Efficacy of Kami Guibi-tang as an Add-On Therapy to Acetylcholinesterase Inhibitor for Cognitive Function in Mild Alzheimer's Disease: A Pilot Study

**DOI:** 10.1155/2023/4846770

**Published:** 2023-01-30

**Authors:** Ha-Ri Kim, Hee-Yeon Shin, Tae-Bin Yim, Geon-Ho Jahng, Chul Jin, Seungwon Kwon, Seung-Yeon Cho, Seong-Uk Park, Woo-Sang Jung, Sang-Kwan Moon, Chang-Nam Ko, Jung-Mi Park

**Affiliations:** ^1^Department of Clinical Korean Medicine, Graduate School, Kyung Hee University, Seoul, Republic of Korea; ^2^Department of Radiology, Kyung Hee University College of Medicine, Kyung Hee University Hospital at Gangdong, Seoul, Republic of Korea; ^3^Department of Cardiology and Neurology, College of Korean Medicine, Kyung Hee University, Seoul, Republic of Korea; ^4^Stroke and Neurological Disorders Center, Kyung Hee University College of Korean Medicine, Kyung Hee University Hospital at Gangdong, Seoul, Republic of Korea

## Abstract

**Background:**

Kami Guibi-tang (KGT), a traditional Korean herbal medicine is mainly used to treat insomnia and nervousness. Acetylcholinesterase inhibitors (AChEIs) are the main treatments for mild Alzheimer's disease (AD), a degenerative brain disease. However, currently no drug can fundamentally treat AD or reverse the advanced cognitive decline. This clinical study explored the efficacy and safety of adding KGT to AChEI for cognitive function in mild AD.

**Methods:**

This was a pilot study for a larger randomized, double-blind, placebo-controlled trial. Participants between 55–90 years diagnosed with mild AD were recruited from Kyung Hee University Hospital at Gangdong, Seoul, Korea. They were randomized to receive either KGT or placebo for 24 weeks, in addition to their regular AChEI. The primary outcome was treatment efficacy, as assessed by the relative amount of change over the study period in total scores on the Dementia version of the Seoul Neuropsychological Screening Battery (SNSB-D). Changes in SNSB subscores were assessed as secondary outcomes. Safety parameters, including adverse events and abnormalities in blood tests, electrocardiograms, and brain magnetic resonance imaging were also monitored.

**Results:**

Between March 2018 and November 2020, seven participants each in the KGT group and the placebo group completed the 24-week trial. There were no significant changes in SNSB-D total or subindex scores for either group (*p* = 0.69 and 0.63, respectively), and no significant differences were observed between them (*p*=0.71). No adverse events related to KGT were reported. We also compared and analyzed the results of a previous pilot study conducted on amnestic mild cognitive impairment (aMCI) using protocol of this study. The aMCI group showed a significant improvement in the total SNSB-D score, especially in the memory domain, compared to the mild AD group (*p* = 0.04 and 0.02, respectively). The Korean Mini-Mental State Exam and Korean Instrumental Activities of Daily Living scores also significantly improved in the aMCI group (*p* = 0.01 and 0.02, respectively).

**Conclusions:**

Compared to placebo, adding KGT to AChEI did not significantly improve cognitive function in SNSB in patients with mild AD. We suggest that KGT would have a positive effect on patients with early stages of cognitive impairment such as aMCI. The findings could assist design larger, longer-term clinical trials of KGT use in elderly patients with mild AD. This study was registered in the Korean Clinical Trial Registry on December 26, 2017, with the CRIS approval number KCT0002904.

## 1. Introduction

Alzheimer's disease (AD) accounts for 60–80% of all cases of dementia [1]. AD is a chronic progressive neurodegenerative disease characterized by memory impairment, gradual deterioration of other cognitive functions, and eventual loss of the ability to perform the functions of daily life. Thus, it affects the well-being of individuals in very profound ways [2].

The number of dementia patients among people aged 65 years and older in Korea has been estimated at 860,000 as of 2019, or approximately 11% of the population. The total number of affected people is expected to exceed one million by 2024 and further to three million by 2050 [3]. The annual economic loss due to dementia is estimated at over 800 billion dollars worldwide, which is larger than that of any other degenerative brain disease [4]. Dementia also has a serious impact on the mental health and quality of life of family members. Therefore, early diagnosis and treatment is important to enable patients and their families to reduce avoidable medical expenses and prepare for various obstacles caused by dementia [3].

The pathogenesis of AD has not yet been fully elucidated. Aggregation of amyloid beta (A*β*) and tau protein in the brain have been proposed as pathological biomarkers for AD [5]. A recent study showed that severely reactive astrocytes contribute to the development of AD [6]. However, no drugs are currently available that can fundamentally treat AD or help patients recover from advanced cognitive decline. Available drugs are mainly used to slow the progression of the disease and maintain cognitive function to the extent possible. In Korea, drugs approved for AD include acetylcholinesterase inhibitors (AChEIs), such as donepezil, rivastigmine, and galantamine, and N-methyl-D-aspartate (NMDA) receptor antagonists, such as memantine. However, in some patients, these drugs cause gastrointestinal side effects, such as nausea, diarrhea, and vomiting [7]. To overcome the limitations of the existing treatments, studies with various complementary and alternative therapies are being undertaken. These include use of vitamin E [8], ginkgo biloba extract [9], and choline alfoscerate [10], which have been tested for treatment of dementia. However, there are no clear indications supporting their efficacy.

Kami Guibi-tang (KGT) is a traditional herbal medicine used in East Asia that is widely used for insomnia and depression [11]. Complementary and alternative medicine (CAM) has been widely used in countries around the world, and herbal medicine is one of the most popular therapy methods in the CAM. The use of CAM in different diseases has been increasing [12], and other clinical trials have shown the efficacy of CAM for different diseases [13–16]. Experimental studies undertaken with KGT have reported that it increases the activity of central nerve cells [17] and improves cognitive impairment by reducing neuronal apoptosis and A*β* accumulation in the hippocampus [18]. It is speculated that the effect of KGT on cognitive function occurs through the reversal of degenerative axonal atrophy and nerve damage caused by the phosphorylation of A*β* and Tau proteins [19–21]. An earlier clinical trial reported that KGT improved cognitive function in patients with mild AD [22]. A combination therapy of KGT and AChEI donepezil over a 16-week period was documented to prolong the effect of donepezil [23], and a crossover study reported that it improved cognitive function [24]. A previous pilot study of KGT in patients with a predementia condition known as amnestic mild cognitive impairment (aMCI) found that KGT had significant efficacy in improving memory [25].

As described above, KGT had a positive effect on the cognitive function of patients with AD. However, previous studies have limitations in that they were not blinded. Additionally, to date, there has been no study that strictly confirmed the efficacy and safety of KGT through randomized, placebo-controlled, and double-blind studies. Therefore, the aim of this pilot study was to rigorously investigate the efficacy and safety of KGT in improving cognitive function in patients with mild AD.

## 2. Materials and Methods

### 2.1. Study Design

This study was designed as a pilot for a randomized, double-blind, placebo-controlled clinical trial, the protocol for which has been published [26]. The study was conducted between March 2018 and November 2020 at the Kyung Hee University Hospital at Gangdong, Seoul, Republic of Korea. During a screening period of approximately 2-weeks from the time the prospective participant visited, he/she was evaluated to determine whether they met the criteria for inclusion in the study. All the patients were treated for a period of 24 weeks and followed-up thereafter for 4 weeks.

We recruited persons who visited our hospital with complaints of impaired memory. Potential participants voluntarily completing a written informed consent form were screened over a 2-week period according to the inclusion/exclusion criteria (as given below), the Korean Mini-Mental State Examination (K-MMSE), and the Korean Dementia Screening Questionnaire (KDSQ). In the inclusion criteria, the continuous use of AChEI medications was stipulated. Potentially eligible participants completed the Seoul Neuropsychological Screening Battery (SNSB), and those diagnosed with mild AD based on their scores by a neurologist were enrolled in the trial.

Participants were randomly allocated to the treatment or control groups. The treatment group received KGT granules, and the control group received placebo granules three times a day for 24 weeks. The dosage of the AChEI medications was maintained in both the groups during the study period. After 24 weeks, the efficacy and safety of the KGT were assessed through comparisons with the baseline. Adverse events were assessed once a week during the medication period and four weeks following the completion of the medication course.

The changes in the Seoul Neuropsychological Screening Battery-Dementia Version (SNSB-D) total scores and the scores of the five SNSB-D domains were compared before and after the 24-week treatment/placebo period as the primary outcome. We considered the SNSB subtest scores and scores on the K-MMSE, Short Geriatric Depression Scale (SGDS), Barthel Index of Activities of Daily Living (Barthel ADL), Korean Instrumental Activities of Daily Living (K-IADL), Clinical Dementia Rating (CDR), and Global Deterioration Scale (GDS) as secondary outcomes. In addition, the changes in the Korean version of Quality of Life-Alzheimer's Disease (KQoL-AD) and Caregiver-Administered Neuropsychiatric Inventory (CGA-NPI) scores were compared. We monitored the safety of KGT by regular checks for adverse events and abnormal findings on vital signs, blood tests, electrocardiogram (ECG), and brain magnetic resonance imaging (MRI).

The study protocol was approved by the Korean Ministry of Food and Drug Safety (MFDS approval number: 31234) as well as the Institutional Review Board of Kyung Hee University Hospital at Gangdong (IRB approval number: KHNMCOH 2017-11-002-001) and was registered in the Clinical Research Information Service (CRIS approval number: KCT0002904). All participants received a sufficient explanation of the background, method, potential risks and benefits, and provisions for confidentiality before the start of the study following, which they voluntarily filled out the written consent form. All the processes of this clinical study were conducted in compliance with the ethical regulations of the Declaration of Helsinki (South Africa Amendment, 1996) and the Korea Good Clinical Practice (KGCP) guidelines. This clinical study complied with the Consolidated Standards of Reporting Trials (CONSORT) statement (Table S2).

### 2.2. Participants

#### 2.2.1. Inclusion Criteria

Participants meeting the following criteria were eligible to participate if they were aged 55–90 years old; complained of impaired memory; were diagnosed with mild AD by a neurologist, based on scores on the SNSB and the criteria of the National Institute of Neurological and Communicative Disorders and Stroke and Alzheimer's Disease and Related Disorders Association (NINCDS-ADRDA), with a CDR of 0.5∼1 point; were constantly taking AChEIs, such as donepezil, rivastigmine, and galantamine, and free of adverse events for the previous four weeks, and cognition-related medications included agents to improve cerebral blood flow and others affecting cognition, such as gliatilin, gliatamin, ginexin, and tanamine; were taking medications such as sleeping pills, antianxiety drugs, antidepressants, antipsychotics, and anticholinergic drugs to stabilize the underlying disease for the previous three months or more, and no indications of probable loss of stability during the 24-week study period; had no problems in communication; no contraindications for MRI.

#### 2.2.2. Exclusion Criteria

Participants who met any of the following criteria were ineligible to participate; a brain disorder causing neurological symptoms other than cognitive dysfunction; formal diagnosis of Parkinson's disease, Huntington's chorea, Down syndrome, Creutzfeldt-Jakob disease, and other neurodegenerative disorders; cognitive impairment resulting from conditions such as head trauma, hypoxic brain damage, vitamin deficiency, brain tumor, encephalitis, neurosyphilis, or mental retardation; cerebrovascular disease documented by MRI; history of convulsive disease other than febrile convulsions in childhood; diagnosed psychiatric disorders such as major depressive disorder, bipolar disorder, schizophrenia, alcoholism, or substance abuse, according to the Diagnostic and Statistical Manual of Mental Disorders, 4^th^ edition (DSM-IV) criteria; life-threatening physical disabilities requiring immediate treatment; uncontrolled hypertension; heart or renal disease; peripheral edema; gastrointestinal symptoms, such as anorexia, stomach discomfort, nausea, abdominal pain, and diarrhea; use of medications that could induce hypokalemia or myopathy; use of NMDA receptor antagonist, such as memantine; hypersensitivity to the medication used in the study; possibility of pregnancy; clinically significant abnormalities in blood chemistry, including levels of serum glutamic pyruvate transaminase (SGPT)/serum glutamic oxaloacetic transaminase (SGOT) being more than twice the normal upper limit, or serum creatinine levels more than 10% above the normal upper limit; participation in any other clinical trial within the previous 4 weeks; illiteracy; considered unsuitable for participation by the investigators.

#### 2.2.3. Termination Criteria

Participants who met any of the following criteria were stopped in the study; severe adverse events making it impossible to continue the clinical trial; individual considered unsuitable for participation by the investigators; voluntary withdrawal; nonobservance of the protocol, i.e., drug compliance below 80%; decision by the principal investigator.

#### 2.2.4. Recruitment

Between March 2018 and May 2020, we recruited patients aged 55‒90 years with complaints of impaired memory through advertisements on bulletin boards, newspapers, and online media. Potential participants were screened using the K-MMSE and the KDSQ. Potentially eligible participants, who completed the SNSB and those who were diagnosed with mild AD by a neurologist, were enrolled in the study.

#### 2.2.5. Randomization and Blinding

Participants were assigned to either the treatment group (KGT group) or control group (placebo group) at a ratio of 1 : 1 using a block randomization method with a block size of 4. The random number table was generated by a researcher who did not participate in the evaluation, and the numbers were assigned sequentially with enrollment. KGT and placebo granules with identical appearance, smell, taste, and properties were produced by the manufacturer (Kyungbang Co., Incheon, Korea). The placebo consisted of corn starch, lactose, hydroxypropyl cellulose, caramel color (food additives), tartrazine (FD and C Yellow 5), Allura Red AC (FD and C Red 40), and Ssanghwa flavor. The manufacturer assembled the products in the medication kits, each labeled with a study-generated random number. An independent pharmacist distributed the kits to the participants in the order of their enrollment. Participants, investigators, pharmacists, and outcome assessors were blinded to the assignment until the end of the trial. The blinding could be broken according to the approved procedure in case of serious adverse events.

### 2.3. Intervention

Participants in both the study groups were instructed to ingest the contents of one 3.0 g packet from their medication, KGT, or placebo granules kit (Kyungbang Co., Incheon, Korea) with water three times a day, 30 min after meals, for 24 weeks. Both drugs were in the form of yellow-brown granules. The components and dosages of KGT are listed in Table 1.

### 2.4. Assessment

#### 2.4.1. Korean Dementia Screening Questionnaire (KDSQ) and Korean Mini-Mental State Examination (K-MMSE)

The KDSQ is a cognitive screening test administered to caregivers of patients [27]. It consists of questions related to memory and behavioral disorders, and problems performing normal daily activities that are common in early AD [28].

The K-MMSE is a simple cognitive function test that evaluates the overall cognitive function and was used to exclude persons with normal cognition, mild cognitive impairment (MCI), or dementia [29]. It is the most widely used cognitive function screening test and is included in the NINCDS-ADRDA diagnostic criteria for AD. Age- and education-specific standard scores have been developed for normal cognitive function.

#### 2.4.2. Seoul Neuropsychological Screening Battery-Dementia Version (SNSB-D)

The SNSB was assessed at baseline and at 24 weeks by an independent clinical psychologist to assess the impact of KGT on cognitive function. The SNSB is a standardized neuropsychological test often used in Korea [30]. This study used the revised version, the SNSB-II, which evaluates five cognitive domains (attention, language ability, memory, spatiotemporal ability, and executive ability) and includes other related tests of cognitive function as subtests [31]. The details have been included in Table S1 in the Supplementary Material.

SNSB-D is a version of the SNSB modified for patients with AD [32]. The test results provide a global cognitive function score, which is the sum of five cognitive domain subtests: attention (17 points, 6%), language ability (27 points, 9%), memory ability (150 points, 50%), spatiotemporal ability (35 points, 12%), and executive ability (70 points, 23%).

The SGDS is a shortened depression scale designed to evaluate depressive symptoms in the elderly [33]. It contains questions covering complaints of memory loss and cognitive dysfunction, which are often the symptoms of depression in the elderly. This scale uses easily understood questions that can be answered with yes or no. The risk of depression increases with the score; in general, scores of 8 (out of 15) or higher indicate a high risk.

The Barthel ADL consists of 10 items that evaluate basic daily activities such as feeding, personal toileting, bathing, dressing and undressing, getting on and off the toilet, controlling bladder, controlling bowel, moving from wheelchair to bed and returning, walking on a level surface or propelling a wheelchair if unable to walk, and ascending and descending stairs [34].

The K-IADL scale evaluates instrumental daily life activities, which are higher than basic daily life activities [35]. It is completed by a caregiver who has witnessed the patient's daily life for at least the previous four weeks and consists of 11 questions on a 0–3 point scale. Topics include shopping, travel (mode of transportation), ability to handle finances, housekeeping (use of electronic devices), preparing food, ability to use the telephone, responsibility for one's own medication, recent memory, hobbies, watching TV, and fixing around the house.

The CDR scale evaluates the overall severity of AD by assessing both the cognitive levels and daily living ability [36]. Six domains of memory, orientation, judgment and problem solving, community affairs, home and hobbies, and personal care were evaluated through semistructured interviews with patients and caregivers. The CDR Global Score (CDR-GS) is based only on the CDR memory score: CDR 0.5, very mild; CDR 1, mild; CDR 2, moderate; CDR 3, severe AD. The CDR sum of boxes (CDR-SB) adds the scores of all six domains with a total score of 30 points. The CDR-SB index can be used to track the degree of response of a patient receiving medication for dementia.

The GDS expresses cognitive decline and subsequent functional deterioration on a scale of 1 to 7 [37]. This scale describes the degree of cognitive impairment at each stage with specific examples and differentiates the initial stages of cognitive impairment in detail. Similar to the CDR, the GDS assesses the severity of dementia and is widely used for clinical research on cognitive drugs and the early diagnosis of dementia.

#### 2.4.3. Korean Version of Quality of Life-Alzheimer's Disease (KQoL-AD)

The KQoL-AD test assesses the overall quality of life of patients with AD [38]. Its reliability and validity are relatively high in patients with mild AD and can be applied to patients with significantly reduced cognitive function. Patients and caregivers separately evaluated physical health, energy level, mood, living situation, memory, relationships with spouse, relatives and friends, self as a whole, chores around the house, leisure activities, financial situation, and life as a whole, each on a scale of 1 to 4.

#### 2.4.4. Caregiver-Administered Neuropsychiatric Inventory (CGA-NPI)

The CGA-NPI is a questionnaire developed to evaluate behavioral disorders in patients with dementia [39]. A caregiver who knows the patient evaluates 12 behavioral disorders: delusions, hallucinations, agitation/aggression, depression/dysphoria, anxiety, elation/euphoria, apathy/indifference, disinhibition, irritability/lability, aberrant motor behavior, sleep/nighttime behavior, and appetite/eating disorders.

#### 2.4.5. Blood Test, Electrocardiogram, and Brain MRI

Blood tests were performed at baseline and at 12 and 24 weeks to evaluate safety. Specifically, blood urea nitrogen, creatinine, SGOT, SGPT, sodium, potassium, chloride, creatine kinase, lactate dehydrogenase, and glucose were monitored to detect abnormalities in liver function, renal function, electrolytes, and heart enzyme levels. Apolipoprotein E (ApoE) genotyping was performed to test for the *ε*4 allele, which carries an increased risk for AD [40]. An ECG was performed at baseline, 12 weeks, and 24 weeks to monitor drug safety. Brain MRI was performed at baseline and after 24 weeks. We checked whether there were any structural abnormalities in the brain through 3D T1 weighted images, 2D T2 weighted images, and fluid-attenuated inversion recovery (FLAIR) images.

### 2.5. Statistical Analysis

#### 2.5.1. Sample Size Calculation

Previous clinical studies reporting on the efficacy of KGT in improving cognitive function in patients with mild AD did not use methodology equivalent to that of the present study in terms of study design and disease state. Thus, there were no available data for calculating the sample size. Since this was a pilot study, the number of study participants was based on the number of patients that could be recruited and the projected cost of the overall study. In general, 20‒40 people per group were suggested as the appropriate number of participants in a pilot study [41]. Accordingly, the target number for this study was set to 30 per group, with the goal of 38 enrolled participants per group to accommodate a dropout rate of up to 20%.

#### 2.5.2. Statistical Analysis

All data were entered into Microsoft Excel 2016 (Microsoft Co., Redmond, USA) and analyzed using SAS statistical software (version 9.4; SAS Institute, Cary, NC, USA). The analysis was performed as intention-to-treat using those who took the test drug at least once after randomization.

Demographic data and cognitive function assessment results, such as the SNSB scores, were tested using the chi-squared test or Fisher's exact test for differences between groups at baseline and to compare prognostic variables. Differences in representative values were tested by the Wilcoxon rank-sum test. For within-group comparisons of continuous variables before and after the study period, paired tests were performed using the Wilcoxon signed-rank test. Statistical analyses were performed at a significance level of 5%, so a *p* value less than 0.05 was considered statistically significant. All descriptive statistics of the results are expressed as mean ± standard deviation (SD) or median (IQR) for continuous variables and frequency (%) for categorical variables.

## 3. Results

### 3.1. Participants

**Figure 1:**
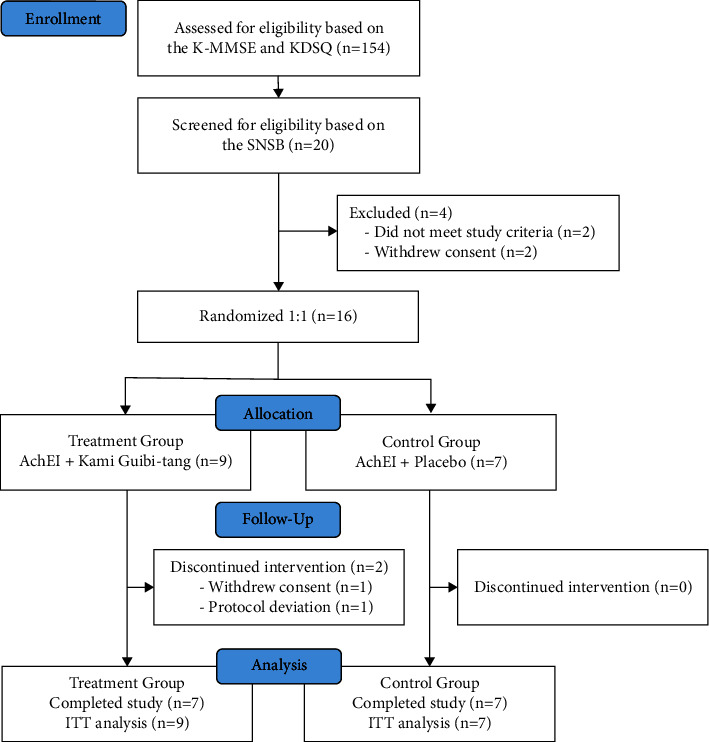
CONSORT diagram. K-MMSE, Korean Mini-Mental State Exam; KDSQ, Korean Dementia Screening Questionnaire; SNSB, Seoul Neuropsychological Screening Battery; AChEI, acetylcholinesterase inhibitor; ITT, intention to treat.

A total of 154 individuals expressed their intention to participate in the study. Prospective participants completed telephone screening and the K-MMSE and KDSQ tests. The SNSB was subsequently administered to 20 potentially eligible individuals whose screening results indicated possible mild AD and who provided written informed consent. Of these 20 patients, two did not meet the inclusion criteria and two withdrew their consent. Based on the SNSB results, 16 participants were diagnosed with mild AD by a neurologist and were enrolled in the study. Among them, nine were randomized to the treatment group and seven to the control group. Two participants in the treatment group did not complete the study; one withdrew and the other had to be excluded from the study due to violation of the study protocol. Both were included in the intention-to-treat analysis. Seven participants each in the treatment and the control group completed the trial **(**Figure 1**)**.

### 3.2. General and Clinical Features

There were no significant differences between the KGT and control participants in terms of the general characteristics such as age, sex, education level, and presence of the ApoE *ε*4 allele at baseline. The results of the SNSB-D and other cognitive tests were not significantly different between the two groups at baseline. However, there was a significant difference in mean SGDS scores, with scores of 6.29 *±* 3.68 and 1.83 ± 2.64 for the KGT and control groups, respectively (*p* = 0.04, Table 2).

### 3.3. Efficacy Outcome

#### 3.3.1. SNSB-D

While the mean score for the SNSB-D in the KGT group showed a nonsignificant decrease from 119.86 ± 30.38 at baseline to 112.93 ± 31.58 at 24 weeks (*p* = 0.69), the score in the control group showed a nonsignificant increase from 93.79 ± 23.58 at baseline to 96.57 ± 19.44 at 24 weeks (*p* = 0.63). The changes in the mean scores were not significantly different between the two groups (*p* = 0.71). Analysis of the changes in the scores in the five domains of the SNSB-D showed no significant differences either within or between the groups (Table 3).

#### 3.3.2. Other Indexes (K-MMSE, SBDS, Barthel ADL, K-IADL, CDR, and GDS)

The K-MMSE scores dropped significantly from baseline to 24 weeks for both the KGT group (22.86 ± 3.67 to 20.29 ± 3.50, *p* = 0.03) and the control group (21.00 ± 2.71 to 17.57 ± 3.82, *p* = 0.03). There were no significant intergroup differences in the amount of change (*p* = 0.80). No significant difference in the SGDS, Barthel-ADL, K-IADL, CDR, or GDS scores were noted either within or between the two groups (Table 4).

#### 3.3.3. KQoL-AD

Scores on the KQoL-AD did not show a significant change in the KGT group, either when the patient self-evaluated (31.14 ± 5.58 to 28.57 ± 4.86, *p* = 0.16) or when the caregiver provided the evaluation (27.86 ± 5.11 to 27.00 ± 7.48, *p* = 0.58). Changes between baseline and 24 weeks in the control group were not significant when the patient self-evaluated (34.29 ± 6.60 to 34.14 ± 5.21, *p* = 1.00) or when the caregiver provided the evaluation (30.57 ± 7.52, 29.86 ± 4.56, *p* = 1.00). There were no significant differences between the treatment and control groups for either the caregiver or patient evaluations (*p* = 0.61 and *p* = 0.66, respectively, Table 4).

#### 3.3.4. CGA-NPI

There was no significant change between baseline and 24 weeks in the CGA-NPI scores in the KGT group (6.86 ± 5.30 to 11.14 ± 8.84, *p* = 0.19) or in the control group (7.00 ± 8.14 to 9.86 ± 12.62, *p* = 0.72). The mean changes too showed no significant intergroup differences (*p* = 1.00; Table 4).

### 3.4. Comparative Analysis with aMCI Group

A pilot study was conducted on aMCI with the same basic protocol as in the present study [25]. With the consent of the author, we compared the data from that study on patients with aMCI receiving KGT (*n* = 16) for 24 weeks with the present data-patients with mild AD receiving KGT (*n* = 7). The SNSB-D total score and memory domain score were significantly higher in the aMCI group than in the mild AD group (*p* = 0.04 and 0.02, respectively). In addition, the Rey recall score in the memory domain, contrasting program score, Luria loop score, and word fluency (animal naming) scores in the frontal and executive function domains showed a significant difference (*p* = 0.03, 0.03, 0.05, and 0.04, respectively). There was no significant difference between the two groups in the scores of the other subtests (Table 5). The amount of change was significantly different between the aMCI and mild AD groups for the K-MMSE and K-IADL scores (*p* = 0.01 and 0.02, respectively). No significant differences between the two groups with respect to the SGDS, Barthel-ADL, GDS, and CDR scores were observed (Table 6).

### 3.5. Safety Outcome

#### 3.5.1. Adverse Events

No adverse events were reported in the KGT group. Though one case of skin rash was reported from among the controls; the severity was very mild and there was spontaneous improvement within a few days after onset.

#### 3.5.2. Other Examinations

There were no significant changes in vital signs, blood tests, ECG, or brain MRI. No clinically significant abnormalities were observed in either group.

## 4. Discussion

To our knowledge, this is the first randomized, double-blind, placebo-controlled study to explore the efficacy of KGT in improving the cognitive function and safety of patients with mild AD. We expanded the methods used in previous studies of KGT to include SNSB, KQoL-AD, and CGA-NPI.

During the 24-week treatment period, changes in the SNSB-D and other indices were not significantly different between the treatment and placebo groups. The SNSB covers a wide range of difficulty levels, allowing an in-depth evaluation of each cognitive domain. However, the test takes about 2 h, which can be a burden for elderly people. Participants experiencing severe cognitive decline, are elderly, or who are uncooperative will have more difficulty completing the test [42]. In this study, the SNSB-D score tended to decrease over time in the KGT group. This is likely a reflection of several participants in the KGT group with high initial scores for negative items in the CGA-NPI, such as apathy/indifference and depression/discouragement.

The mean K-MMSE scores decreased significantly over the study period in both groups, although the comparison between them was not significant. Most studies on mild AD have used MMSE-1 as an evaluation tool [22–24]. On an average, the MMSE-1 scores in patients with mild AD decrease by 3.8 points per year [43]. In previous KGT treatment studies, the MMSE-1 score was maintained or increased by an average of 1 point. In contrast, it has been reported that among patients with mild AD, higher initial MMSE-1 scores are associated with lower drug treatment results [44]. The initial MMSE-1 scores of this study averaged 21–22 points, higher than those reported for clinical studies of KGT [22, 24], and this difference in cognitive levels as measured by the MMSE-1 may have influenced the treatment efficacy.

Additionally, we compared the effects of KGT on the cognitive function of patients with aMCI and mild AD and analyzed the differences between the two groups. According to a previous preliminary clinical study [25], the KGT treatment group with aMCI showed a significant improvement in the SNSB-D score. In this study, compared to the mild AD group, KGT was associated with a significant improvement in the SNSB-D score in the MCI stage than in the mild AD stage, particularly in the memory domain of SNSB-D. The results showed that KGT significantly improved the Rey recall score in the memory domain, contrasting program, the Luria loop, and the word fluency score in the frontal and executive function domains showed significant differences.

The Rey recall test is a memory recall test belonging to the Rey-Osterrieth Complex Figure Test, a representative test tool for spatiotemporal cognitive function, and checks whether the stimulus is accurately input by the patient as they must draw the same as the given picture stimulus, by remembering that was viewed. It is also a useful tool for evaluating the executive function related to frontal lobe function by redrawing it. In patients with mild AD, the Rey recall score of the SNSB is related to brain gray matter volume, which is known to be associated with impaired memory of language and visual information due to atrophy of the superior temporal gyrus [45]. The contrasting program test evaluates the ability to control movements and learn rules. If the tester raised two fingers, the subject was instructed to raise one finger, and if the tester raised one finger, the subject was instructed to raise two fingers. If frontal lobe dysfunction is severe, the subject follows the examiner's finger. In this case, if response inhibition is not performed, it is judged that there is defective response inhibition [46]. The Luria loop test is used to determine if there is internal perseveration by drawing several loops with three rings [47]. The word fluency test, the animal naming, evaluates semantic word fluency by making the animal's name words spoken as much as possible within 1 min. Semantic fluency is related to semantic memory, which is applied to words and meanings, and to components of long-term memory. The impairment of the semantic memory test means there is a lack of access to item knowledge or loss of representational knowledge [48]. In general, it is known that frontal lobe function is maintained in the early stages of AD, but studies have shown that frontal executive function abnormalities appear at an early stage [49]. Executive function is concerned with the control and regulation of cognitive processes and goal-directed, future-oriented behavior [50].

The significant improvement in the memory and frontal function test scores in the aMCI group showed that KGT was more effective in the MCI stage than in the mild AD stage. It is thought that KGT has the potential to have a positive effect on neuropathology that can progress to AD, such as temporal lobe atrophy, as well as improving memory, language, and visual information processing ability. However, we found that KGT was effective in improving cognitive function in patients with aMCI but not in those with mild AD. Therefore, it is necessary to consider the reasons for these differences. First, participants in the aMCI pilot study took KGT alone, whereas our study participants took KGT with AChEI. These conventional drugs may have masked the effects of KGT, but discontinuing an AChEI to participate in a study creates an ethical problem. Second, because aMCI is a predementia stage before neurodegenerative changes have occurred, the efficacy of KGT may have been better expressed at this stage than at the stage of mild AD, in which the degenerative changes have already begun, and recovery cannot be expected. Finally, approximately 20% of patients with aMCI naturally improve to normal [51]. It is remarkable that KGT showed significant effects on cognitive function improvement in patients with aMCI before the treatment guidelines for KGT in MCI have been established. Therefore, these results indicate the need for initiating KGT treatment at earlier stages of cognitive impairment.

The effects of KGT have been demonstrated in experimental studies in mice. In a 2008 study on an A*β*-induced mouse model of AD, ingestion of Guibi-tang for 3 days improved memory acquisition, memory retention, and object recognition [19]. In a 2011 5FXAD mouse study, KGT improved object recognition and reduced the number of amyloid plaques in the frontal cortex and hippocampus [20]. In another study, KGT ameliorated A*β*-induced tau phosphorylation and axonal atrophy [21]. Previous clinical studies testing the effects of KGT on cognitive function include clinical trials and retrospective chart reviews. All studies used the MMSE-1 as an evaluation tool. In a clinical trial reported by Higashi et al., the MMSE-1 score was significantly higher than that in the nontreatment group and the group receiving Goshajinkigan [22]. A crossover test by Watari et al., reported that MMSE-1 scores were significantly increased after 16 weeks of combination therapy with donepezil compared to 16 weeks of donepezil alone [23]. In a retrospective study, the MMSE-1 scores were lower in patients receiving donepezil alone than in those receiving donepezil plus KGT [24].

KGT is derived from 15 herbs and plants. Studies have documented positive effects on the cognitive function of each component. *Polygalae radix*, the main ingredient of KGT, acts on the cholinergic system of the CNS66, and exerts effects on neurodegenerative pathologies through an increase in acetylcholine levels, such as improving cognitive function, neuroprotection against A*β*, and suppression of neuroinflammation [52]. *Gardeniae fructus*, another key ingredient, promotes cholinergic neurotransmission and improves memory [53]. *Moutan cortex* inhibits the aggregation of A*β* proteins [54]. *Poria sclerotium* is a long-term synergistic effector of the hippocampus [55] and inhibits acetylcholinesterase [56]. *Zizyphi fructus* reduced the number of activated microglia and astrocytes observed after A*β* injection [57]. This combination of properties clarifies the possible mechanisms of KGT efficacy.

This study has several limitations. First, we were able to recruit only fewer participants than planned. This is partly due to the inclusion criteria, which were stricter than those in previous studies. In addition, the latter part of the study period overlapped with the COVID-19 pandemic, making it more difficult to recruit participants. The resulting small sample size led to low statistical power, affecting our ability to detect significant differences. Several of the limitations mentioned below reflect the small sample size. Second, despite the use of randomization to minimize disturbance bias, an imbalance in the SGDS score occurred at baseline. Specifically, participants taking antidepressants were assigned to the KGT group. It is known that 30%–50% of patients with AD suffer from depression [58], but depression is not the only risk factor for cognitive decline. The relationship between depression and AD is complex; the conditions may appear independently, or depression may appear as a secondary reaction to cognitive impairment. Since antidepressant medications can affect cognitive function, the imbalance in assignment may have affected the results. Three participants with SGDS scores of 8 or higher were assigned to the KGT group, and two of them improved to the normal range after treatment. Third, AD was only diagnosed by SNSB at the screening stage, rather than by imaging tests such as amyloid PET, tau PET, or neurodegeneration MRI. Fourth, the SNSB-D score tended to decrease in the KGT group. There were several participants with initial high apathy/indifference and agitation/aggression scores on the CGA-NPI. These participants were uncooperative and complained of difficulty completing longer tests such as the SNSB. Fifth, this study examined KGT as an add-on therapy for AChEI and could not assess its effects when used alone. The previous aMCI pilot study used KGT alone and found efficacy in improving cognitive function. Finally, the study was not designed with a follow-up observation period, so any long-term effects of the treatment on disease progression could not be determined.

This study was a productive first attempt to observe the efficacy and safety of KGT in mild AD, which remains incurable. The results of this study can be used as reference data for future large-scale clinical trials on the efficacy of KGT for treating cognitive diseases.

## 5. Conclusions

KGT treatment over 24 weeks did not have a significant impact on overall cognitive function as measured by the SNSB in patients with mild AD. KGT was associated with significant improvements in the memory domain in the aMCI group compared to the mild AD group, and no adverse events were reported. The results of this pilot study will be valuable in designing future larger-scale clinical trials on the efficacy of KGT for treating cognitive diseases.

## Figures and Tables

**Table 1:** Composition of Kami Guibi-tang.

Scientific name	Latin name	Amount (g)
*Panax ginseng* C. A. Meyer	*Ginseng radix*	1
*Atractylodes macrocephala* Koidzumi	*Atractylodes rhizoma* Alba	1
*Poria cocos* Wolf	*Poria sclerotium*	1
*Astragalus membranaceus* Bunge	*Astragali radix*	1
*Dimocarpus longan* Loureiro	*Longanae arillus*	1
*Zizyphus jujuba* Miller var. *spinosa* Hu ex H. F. Chou	*Zizyphi semen*	1
*Bupleurum falcatum* Linné	*Bupleuri radix*	1
*Angelica gigas* Nakai	*Angelicae gigantis Radix*	0.67
*Polygala tenuifolia* Willdenow	*Polygalae radix*	0.67
*Gardenia jasminoides* Ellis	*Gardeniae fructus*	0.67
*Paeonia suffruticosa* Andrews	*Moutan cortex*	0.67
*Zizyphus jujuba* Miller var. *inermis* Rehder	*Zizyphi fructus*	0.67
*Aucklandia lappa* Decne	*Aucklandiae radix*	0.33
*Glycyrrhiza uralensis* Fischer	*Glycyrrhizae radix* et Rhizoma	0.33
*Zingiber officinale* Roscoe	*Zingiberis rhizoma* Recens	0.33

**Table 2:** Baseline characteristics of participants.

Characteristic		KGT (*n* = 9)	Placebo (*n* = 7)	^†^ *p* value
Age (years)		70.7 ± 6.8, 79.0 (12.0)	75.7 ± 10.1, 69.0 (9.0)	0.18
Sex	Female	6 (85.7%)	5 (71.4%)	1.00
	Male	1 (14.3%)	2 (28.6%)	
Education	Elementary school graduation	3 (42.9%)	3 (42.9%)	1.00
	High school graduation	3 (42.9%)	2 (28.6%)	
	University graduation	1 (14.3%)	2 (28.6%)	
Presence of ApoE *ε*4 allele		1 (14.3%)	1 (14.3%)	1.00
Clinical characteristics				
SNSB-D total score		119.86 ± 30.38, 115.00 (59.00)	93.79 ± 23.58, 86.00 (46.50)	0.18
Attention		2.43 ± 0.53, 7.00 (5.00)	2.57 ± 1.13, 7.00 (2.00)	0.25
Language and related function		18.14 ± 5.11, 18.00 (12.00)	16.43 ± 5.88, 19.00 (8.00)	0.62
Visuospatial function		25.43 ± 10.30, 28.00 (10.50)	27.36 ± 5.38, 31.50 (18.00)	1.00
Memory		36.00 ± 16.56, 41.50 (26.00)	21.57 ± 8.13, 20.00 (14.00)	0.12
Frontal and executive function		33.29 ± 14.16, 33.00 (16.00)	22.29 ± 9.50, 24.00 (16.00)	0.17
Other indexes				
K-MMSE		22.86 ± 3.67, 22.00 (6.00)	21.00 ± 2.71, 21.00 (2.00)	0.53
SGDS		6.29 ± 3.68, 4.00 (5.00)	1.83 ± 2.64, 1.00 (2.00)	0.04
Barthel-ADL		20.00 ± 0.00, 20.00 (0.00)	19.29 ± 1.50, 20.00 (1.00)	0.20
K-IADL		0.65 ± 0.39, 0.56 (0.73)	1.13 ± 0.69, 1.00 (1.06)	0.18
CDR-SB		4.86 ± 1.03, 4.50 (1.00)	5.50 ± 1.58, 5.00 (1.00)	0.26
CDR-GS		1.00 ± 0.00, 1.00 (0.00)	1.00 ± 0.00, 1.00 (1.00)	1.00
GDS		4.71 ± 0.49, 5.00 (1.00)	4.86 ± 0.38, 5.00 (0.00)	0.60
KQoL-AD				
Patients		31.14 ± 5.58, 28.00 (10.00)	34.29 ± 6.60, 35.00 (10.00)	0.42
Caregivers		27.86 ± 5.11, 26.00 (6.00)	30.57 ± 7.52, 33.00 (16.00)	0.75
CGA-NPI		6.86 ± 5.30, 8.00 (12.00)	7.00 ± 8.14, 3.00 (12.00)	0.90

KGT, Kami Guibi-tang; ApoE, apolipoprotein E; SNSB-D, Seoul Neuropsychological Screening Battery-Dementia Version; K-MMSE, Korean Mini-Mental State Examination; SGDS, Short-form Geriatric Depression Scale; Barthel-ADL, Barthel index of activities of daily living; K-IADL, Korean Instrumental Activities of Daily Living; CDR-SB, Clinical Dementia Rating Scale–Sum of Boxes; CDR-GS, Clinical Dementia Rating Scale-Global Score; GDS, Global Deterioration Scale; KQoL-AD, Korean Quality of Life-Alzheimer's disease; CGA-NPI, Caregiver-Administered Neuropsychiatric Inventory. Values are presented as mean ± standard deviation, median (interquartile range), or number (proportions, %). ^†^Based on chi-squared or Fisher's exact test.

**Table 3:** Changes in SNSB scores between baseline and 24th-weekfollow-up.

Variable	KGT (*n* = 9)				Placebo (*n* = 7)				^‡^ *p* value for treatment difference
	Baseline	24th week	Change	^†^ *p* value	Baseline	24th week	Change	^†^ *p* value	
Total SNSB-D score	119.86 ± 30.38, 115.00 (59.00)	112.93 ± 31.58, 108.00 (39.00)	−6.93 ± 27.84, −1.50 (36.50)	0.69	93.79 ± 23.58, 86.00 (46.50)	96.57 ± 19.44, 96.00 (36.50)	2.79 ± 8.43, −1.00 (10.00)	0.63	0.71
Attention	7.00 ± 1.15, 7.00 (2.00)	7.00 ± 2.08, 7.00 (4.00)	0.00 ± 1.53, 0.00 (3.00)	1.00	6.14 ± 2.19, 7.00 (5.00)	7.14 ± 1.07, 7.00 (2.00)	1.00 ± 2.38, 0.00 (4.00)	0.44	0.57
Digit span forward	4.57 ± 0.98, 4.00 (2.00)	4.86 ± 1.21, 5.00 (2.00)	0.29 ± 0.95, 0.00 (1.00)	0.75	3.57 ± 1.72, 4.00 (2.00)	4.43 ± 0.79, 4.00 (1.00)	0.86 ± 1.86, 0.00 (3.00)	0.25	0.80
Digit span backward	2.43 ± 0.53, 2.00 (1.00)	2.14 ± 1.07, 2.00 (1.00)	−0.29 ± 0.95, 0.00 (1.00)	0.75	2.57 ± 1.13, 3.00 (0.00)	2.71 ± 0.49, 3.00 (1.00)	0.14 ± 1.35, 0.00 (1.00)	1.00	0.89
Language and related function	18.14 ± 5.11, 19.00 (8.00)	18.29 ± 4.11, 17.00 (7.00)	0.14 ± 2.79, 0.00 (3.00)	0.88	16.43 ± 5.88, 18.00 (12.00)	17.14 ± 4.78, 19.00 (9.00)	0.71 ± 2.69, 0.00 (3.00)	0.81	1.00
K-BNT	8.86 ± 3.53, 11.00 (6.00)	8.71 ± 3.04, 8.00 (6.00)	−0.14 ± 1.95, 0.00 (2.00)	1.00	7.86 ± 2.85, 9.00 (6.00)	7.86 ± 3.08, 9.00 (5.00)	0.00 ± 1.63, 0.00 (2.00)	1.00	0.80
Calculation	9.29 ± 2.36, 8.00 (4.00)	9.57 ± 2.23, 10.00 (5.00)	0.29 ± 1.25, 0.00 (3.00)	0.50	8.57 ± 3.21, 9.00 (5.00)	9.29 ± 2.14, 10.00 (4.00)	0.71 ± 2.56, 0.00 (2.00)	0.81	1.00
Visuospatial function	25.43 ± 10.30, 31.50 (18.00)	25.71 ± 9.31, 30.00 (9.50)	0.29 ± 6.42, −1.50 (9.50)	1.00	27.36 ± 5.38, 28.00 (10.50)	25.00 ± 6.06, 27.50 (10.00)	−2.36 ± 6.66, −2.00 (4.50)	0.20	0.66
Memory	36.00 ± 16.56, 41.50 (26.00)	33.07 ± 13.40, 34.50 (26.00)	−2.93 ± 16.99, −2.50 (29.50)	0.72	21.57 ± 8.13, 20.00 (14.00)	24.43 ± 9.55, 27.00 (11.00)	2.86 ± 7.47, 6.00 (11.50)	0.38	0.53
Orientation	4.71 ± 1.89, 6.00 (4.00)	3.86 ± 2.12, 5.00 (4.00)	−0.86 ± 1.07, −1.00 (1.00)	0.13	3.57 ± 1.51, 3.00 (3.00)	3.43 ± 1.40, 4.00 (3.00)	−0.14 ± 1.86, −1.00 (3.00)	1.00	0.60
SVLT recall	13.57 ± 5.50, 15.00 (12.00)	13.57 ± 4.47, 14.00 (5.00)	0.00 ± 7.42, 0.00 (7.00)	0.81	9.43 ± 3.87, 8.00 (7.00)	11.71 ± 5.06, 11.00 (10.00)	2.29 ± 3.04, 3.00 (5.00)	0.16	0.62
SVLT recognition	5.86 ± 4.02, 4.00 (7.00)	5.29 ± 2.69, 5.00 (4.00)	−0.57 ± 4.54, 0.00 (7.00)	0.88	3.14 ± 1.95, 3.00 (4.00)	4.00 ± 2.45, 5.00 (4.00)	0.86 ± 2.97, 1.00 (6.00)	0.52	0.62
Rey recall	6.00 ± 5.07, 7.00 (9.50)	6.36 ± 7.28, 7.50 (9.00)	0.36 ± 7.81, 0.00 (5.00)	0.81	3.57 ± 2.56, 3.50 (4.50)	1.86 ± 1.89, 1.00 (3.50)	−1.71 ± 2.63, −1.50 (3.00)	0.16	0.57
Rey recognition	5.86 ± 4.18, 5.00 (2.00)	4.00 ± 2.94, 4.00 (4.00)	−1.86 ± 4.56, −1.00 (8.00)	0.44	1.86 ± 2.19, 1.00 (5.00)	3.43 ± 4.43, 2.00 (6.00)	1.57 ± 4.89, 1.00 (4.00)	0.47	0.27
Frontal and executive function	33.29 ± 14.16, 33.00 (16.00)	28.86 ± 16.95, 21.00 (25.00)	−4.43 ± 7.52, −1.00 (12.00)	0.25	22.29 ± 9.50, 24.00 (16.00)	22.86 ± 5.21, 23.00 (11.00)	0.57 ± 6.73, 0.00 (9.00)	1.00	0.39
Motor impersistence	3.00 ± 0.00, 3.00 (0.00)	3.00 ± 0.00, 3.00 (0.00)	0.00 ± 0.00, 0.00 (0.00)	—	3.00 ± 0.00, 3.00 (0.00)	3.00 ± 0.00, 3.00 (0.00)	0.00 ± 0.00, 0.00 (0.00)	—	1.00
Contrasting program	2.43 ± 0.98, 3.00 (2.00)	1.43 ± 0.98, 1.00 (1.00)	−1.00 ± 1.15, −1.00 (2.00)	0.13	1.71 ± 1.38, 2.00 (3.00)	2.14 ± 1.07, 3.00 (2.00)	0.43 ± 1.27, 0.00 (1.00)	0.75	0.08
Go-no-go test	1.00 ± 1.00, 1.00 (1.00)	1.29 ± 0.95, 1.00 (1.00)	0.29 ± 0.49, 0.00 (1.00)	0.50	1.00 ± 0.82, 1.00 (2.00)	1.29 ± 0.95, 1.00 (1.00)	0.29 ± 1.11, 0.00 (2.00)	0.75	1.00
Fist-edge-palm	2.43 ± 0.53, 2.00 (1.00)	2.29 ± 0.95, 3.00 (2.00)	−0.14 ± 1.07, 0.00 (2.00)	1.00	1.86 ± 0.90, 2.00 (2.00)	2.43 ± 0.79, 3.00 (1.00)	0.57 ± 0.53, 1.00 (1.00)	0.13	0.21
Luria loop	2.57 ± 1.13, 3.00 (0.00)	1.71 ± 1.60, 3.00 (3.00)	−0.86 ± 1.46, 0.00 (3.00)	0.50	2.14 ± 1.46, 3.00 (3.00)	2.00 ± 1.41, 3.00 (3.00)	−0.14 ± 1.46, 0.00 (0.00)	1.00	0.39
Word fluency: animal	10.14 ± 4.26, 9.00 (4.00)	4.43 ± 5.44, 8.00 (4.00)	−2.43 ± 2.64, −2.00 (4.00)	0.08	7.14 ± 2.19, 8.00 (5.00)	5.71 ± 1.38, 5.00 (2.00)	−1.43 ± 1.62, −1.00 (3.00)	0.13	0.49
Word fluency: Korean	4.86 ± 3.63, 5.00 (5.00)	4.43 ± 5.44, 4.00 (7.00)	−0.43 ± 2.76, 0.00 (3.00)	0.69	2.71 ± 2.81, 1.00 (6.00)	2.71 ± 2.87, 1.00 (5.00)	0.00 ± 3.00, 0.00 (2.00)	1.00	0.75
Stroop test	6.86 ± 4.49, 5.00 (6.00)	7.00 ± 5.72, 6.00 (9.00)	0.14 ± 2.12, 0.00 (4.00)	1.00	2.71 ± 4.27, 2.00 (3.00)	3.57 ± 3.69, 2.00 (7.00)	0.86 ± 4.06, 0.00 (9.00)	0.75	0.90

SNSB-D, Seoul Neuropsychological Screening Battery-Dementia Version; KGT, Kami Guibi-tang, K-BNT; Korean version of Boston Naming Test, SVLT; Seoul Verbal Learning Test. Values are presented as mean ± standard deviation, median (interquartile range). ^†^Based on Wilcoxon signed rank test. ^‡^Based on Wilcoxon rank sum test.

**Table 4:** Changes in SNSB subscores, KQOL-AD, and CGA-NPI scores between baseline and 24th-weekfollow-up.

Variable	KGT (*n* = 9)				Placebo (*n* = 7)				^‡^ *p* value for treatment difference
	Baseline	24th week	Change	^†^ *p* value	Baseline	24th week	Change	^†^ *p* value	
K-MMSE	22.86 ± 3.67, 22.00 (6.00)	20.29 ± 3.50, 20.00 (7.00)	−2.57 ± 1.51, −2.00 (2.00)	0.03	21.00 ± 2.71, 21.00 (2.00)	17.57 ± 3.82, 17.00 (7.00)	−3.43 ± 3.31, −3.00 (2.00)	0.03	0.80
SGDS	6.29 ± 3.68, 4.00 (5.00)	5.00 ± 4.58, 4.00 (6.00)	−1.29 ± 2.29, −1.00 (3.00)	0.25	1.83 ± 2.64, 1.00 (2.00)	1.43 ± 1.90, 1.00 (3.00)	−0.67 ± 3.61, −0.50 (2.00)	0.81	0.62
Barthel-ADL	20.00 ± 0.00, 20.00 (0.00)	19.71 ± 0.76, 20.00 (0.00)	−0.29 ± 0.76, 0.00 (0.00)	1.00	19.29 ± 1.50, 20.00 (1.00)	19.43 ± 0.79, 20.00 (1.00)	0.14 ± 1.86, 0.00 (1.00)	1.00	1.00
K-IADL	0.65 ± 0.39, 0.56 (0.73)	0.86 ± 0.44, 0.80 (0.72)	0.20 ± 0.29, 0.11 (0.61)	0.09	1.13 ± 0.69, 1.00 (1.06)	1.00 ± 0.62, 1.11 (1.04)	−0.12 ± 0.65, −0.11 (0.49)	0.50	0.20
CDR-GS	1.00 ± 0.00, 1.00 (0.00)	0.93 ± 0.19, 1.00 (0.00)	−0.07 ± 0.19, 0.00 (0.00)	1.00	1.00 ± 0.00, 1.00 (0.00)	1.00 ± 0.00, 1.00 (0.00)	0.00 ± 0.00, 0.00 (0.00)	—	0.41
CDR-SB	4.86 ± 1.13, 4.50 (1.00)	5.00 ± 1.61, 5.00 (2.50)	0.14 ± 0.69, 0.00 (1.50)	0.53	5.50 ± 1.58, 5.00 (1.00)	5.86 ± 0.69, 6.00 (1.00)	0.36 ± 1.28, 1.00 (2.00)	0.56	0.45
GDS	4.71 ± 0.49, 5.00 (1.00)	4.71 ± 0.49, 5.00 (1.00)	0.00 ± 0.00, 0.00 (0.00)	—	4.86 ± 0.38, 5.00 (0.00)	4.86 ± 0.38, 5.00 (0.00)	0.00 ± 0.00, 0.00 (0.00)	—	1.00
KQOL-AD									
Patients	31.14 ± 5.58, 28.00 (10.00)	28.57 ± 4.85, 29.00 (9.00)	−2.57 ± 4.42, −2.00 (4.00)	0.16	34.29 ± 6.60, 35.00 (10.00)	34.14 ± 5.21, 32.00 (9.00)	−0.14 ± 8.93, −2.00 (15.00)	1.00	0.61
Caregivers	27.86 ± 5.11, 26.00 (6.00)	27.00 ± 7.48, 25.00 (11.00)	−0.86 ± 5.24, −3.00 (8.00)	0.58	30.57 ± 7.52, 33.00 (16.00)	29.86 ± 4.56, 29.00 (7.00)	−0.71 ± 5.44, 1.00 (8.00)	1.00	0.66
CGA-NPI	6.86 ± 5.30, 8.00 (12.00)	11.14 ± 8.84, 10.00 (9.00)	4.29 ± 6.34, 2.00 (9.00)	0.19	7.00 ± 8.14, 3.00 (12.00)	9.86 ± 12.62, 4.00 (12.00)	2.86 ± 14.95, 3.00 (24.00)	0.72	1.00

KGT, Kami Guibi-tang; SNSB-D, Seoul Neuropsychological Screening Battery-Dementia Version; K-MMSE, Korean Mini-Mental State Examination; SGDS, Short Geriatric Depression Scale; Barthel-ADL, Barthel Activities of Daily Living; K-IADL, Korean-Instrumental Activities of Daily Living; CDR-SB, Clinical Dementia Rating Scale–Sum of Boxes; CDR-GS, Clinical Dementia Rating Scale-Global Score; GDS, Global Deterioration Scale; KQoL-AD, Korean version of Quality of Life-Alzheimer's disease; CGA-NPI, Caregiver-Administered Neuropsychiatric Inventory. Values are presented as mean ± standard deviation, median (interquartile range). ^†^Based on Wilcoxon signed rank test. ^‡^Based on Wilcoxon rank sum test.

**Table 5:** Changes in SNSB-D score between baseline and 24th-weekfollow-up with the aMCI group.

Variable	Mild AD (*n* = 7)				aMCI (*n* = 16)				^‡^ *p* value for treatment difference
	Baseline	24th week	Change	^†^ *p* value	Baseline	24th week	Change	^†^ *p* value	
Total SNSB-D score	119.86 ± 30.38, 115.00 (59.00)	112.93 ± 31.59, 108.00 (39.00)	−6.93 ± 27.84, −1.50 (36.50)	0.69	176.00 ± 24.76, 175.25 (33.25)	198.66 ± 31.25, 200.75 (49.25)	22.66 ± 20.22, 21.75 (31.50)	**<0.01**	**0.04**
Attention	7.00 ± 1.15, 7.00 (2.00)	7.00 ± 2.08, 7.00 (4.00)	0.00 ± 1.53, 0.00 (3.00)	1.00	9.50 ± 2.25, 10.00 (3.00)	10.25 ± 2.32, 11.00 (3.00)	0.75 ± 1.73, 0.50 (2.00)	0.13	0.44
Digit span forward	4.57 ± 0.98, 4.00 (2.00)	4.86 ± 1.21, 5.00 (2.00)	0.29 ± 0.96, 0.00 (1.00)	0.75	5.69 ± 1.25, 6.00 (2.00)	6.06 ± 1.48, 6.00 (2.50)	0.38 ± 1.59, 0.00 (2.00)	0.44	0.81
Digit span backward	2.43 ± 0.53, 2.00 (1.00)	2.14 ± 1.07, 2.00 (1.00)	−0.29 ± 0.95, 0.00 (1.00)	0.75	3.81 ± 1.17, 4.00 (1.00)	4.19 ± 1.38, 4.00 (1.00)	0.38 ± 0.81, 0.00 (1.00)	0.15	0.15
Language and related function	18.14 ± 5.11, 19.00 (8.00)	18.29 ± 4.11, 17.00 (7.00)	0.14 ± 2.79, 0.00 (3.00)	0.88	23.63 ± 2.31, 23.50 (2.50)	23.88 ± 2.25, 24.50 (4.00)	0.25 ± 1.48, 0.00 (2.00)	0.48	0.92
K-BNT	8.86 ± 3.53, 11.00 (6.00)	8.71 ± 3.04, 8.00 (6.00)	−0.14 ± 1.95, 0.00 (2.00)	1.00	12.50 ± 1.63, 12.50 (2.50)	12.38 ± 1.78, 12.50 (3.00)	−0.13 ± 1.31, 0.00 (1.50)	0.85	0.73
Calculation	9.29 ± 2.36, 8.00 (4.00)	9.57 ± 2.23, 10.00 (5.00)	0.29 ± 1.25, 0.00 (3.00)	0.50	11.13 ± 1.54, 12.00 (1.50)	11.50 ± 1.15, 12.00 (1.00)	0.38 ± .062, 0.00 (1.00)	0.06	0.55
Visuospatial function	25.43 ± 10.30, 31.50 (18.00)	25.71 ± 9.31, 30.00 (9.50)	0.29 ± 6.42, −1.50 (9.50)	1.00	32.94 ± 2.82, 34.00 (4.50)	33.81 ± 1.42, 34.00 (2.50)	0.88 ± 3.29, 1.00 (5.50)	0.46	0.46
Memory	36.00 ± 16.56, 41.50 (26.00)	33.07 ± 13.40, 34.50 (26.00)	−2.93 ± 16.99, −2.50 (29.50)	0.72	57.88 ± 17.76, 59.50 (16.75)	74.34 ± 22.66, 72.50 (31.75)	16.47 ± 12.19, 17.50 (17.50)	**<0.01**	**0.02**
Orientation	4.71 ± 1.89, 6.00 (4.00)	3.86 ± 2.12, 5.00 (4.00)	−0.86 ± 1.07, −1.00 (1.00)	0.13	5.88 ± 0.34, 6.00 (0.00)	5.69 ± 0.60, 6.00 (0.50)	−0.19 ± 0.66, 0.00 (0.00)	0.50	0.090
SVLT recall	13.57 ± 5.50, 15.00 (12.00)	13.57 ± 4.47, 14.00 (5.00)	0.00 ± 7.42, 0.00 (7.00)	0.81	19.56 ± 5.64, 17.50 (9.50)	23.63 ± 7.47, 24.50 (11.50)	4.06 ± 4.54, 4.00 (7.00)	**<0.01**	0.28
SVLT recognition	5.86 ± 4.02, 4.00 (7.00)	5.29 ± 2.69, 5.00 (4.00)	−0.57 ± 4.54, 0.00 (7.00)	0.88	6.56 ± 2.58, 7.00 (2.00)	7.50 ± 2.76, 7.50 (4.50)	0.94 ± 2.49, 1.50 (2.50)	0.12	0.64
Rey recall	6.00 ± 5.07, 7.00 (9.50)	6.36 ± 7.28, 7.50 (9.00)	0.36 ± 7.81, 0.00 (5.00)	0.81	20.00 ± 10.30, 20.00 (14.00)	29.91 ± 13.54, 29.50 (20.00)	9.91 ± 7.62, 9.75 (11.50)	**<0.01**	**0.03**
Rey recognition	5.86 ± 4.18, 5.00 (2.00)	4.00 ± 2.94, 4.00 (4.00)	−1.86 ± 4.56, −1.00 (8.00)	0.44	5.88 ± 2.55, 6.50 (3.50)	7.63 ± 1.89, 8.00 (2.50)	1.75 ± 1.91, 2.00 (2.00)	**<0.01**	0.06
Frontal and executive function	33.29 ± 14.16, 33.00 (16.00)	28.86 ± 16.95, 21.00 (25.00)	−4.43 ± 7.52, −1.00 (12.00)	0.25	52.06 ± 9.23, 55.00 (15.50)	56.38 ± 8.38, 58.00 (8.50)	4.31 ± 10.68, 4.00 (11.00)	0.109	0.06
Motor impersistence	3.00 ± 0.00, 3.00 (0.00)	3.00 ± 0.00, 3.00 (0.00)	0.00 ± 0.00, 0.00 (0.00)	—	3.00 ± 0.00, 3.00 (0.00)	3.00 ± 0.00, 3.00 (0.00)	0.00 ± 0.00, 0.00 (0.00)	—	1.00
Contrasting program	2.43 ± 0.98, 3.00 (2.00)	1.43 ± 0.98, 1.00 (1.00)	−1.00 ± 1.15, −1.00 (2.00)	0.13	2.81 ± 0.54, 3.00 (0.00)	2.88 ± 0.34, 3.00 (0.00)	0.06 ± 0.68, 0.00 (0.00)	1.00	**0.03**
Go-no-go test	1.00 ± 1.00, 1.00 (1.00)	1.29 ± 0.95, 1.00 (1.00)	0.29 ± 0.49, 0.00 (1.00)	0.50	2.31 ± 0.87, 3.00 (1.50)	2.44 ± 0.89, 3.00 (1.50)	0.13 ± 1.09, 0.00 (0.50)	0.81	0.73
Fist-edge-palm	2.43 ± 0.53, 2.00 (1.00)	2.29 ± 0.95, 3.00 (2.00)	−0.14 ± 1.07, 0.00 (2.00)	1.00	2.94 ± 0.25, 3.00 (0.00)	3.00 ± 0.00, 3.00 (0.00)	0.06 ± 0.25, 0.00 (0.00)	1.00	0.86
Luria loop	2.57 ± 1.13, 3.00 (0.00)	1.71 ± 1.60, 3.00 (3.00)	−0.86 ± 1.46, 0.00 (3.00)	0.50	3.00 ± 0.00, 3.00 (0.00)	3.00 ± 0.00, 3.00 (0.00)	0.00 ± 0.00, 0.00 (0.00)	—	**0.05**
Word fluency: animal	10.14 ± 4.26, 9.00 (4.00)	7.71 ± 3.45, 8.00 (4.00)	−2.43 ± 2.64, −2.00 (4.00)	0.08	14.06 ± 4.02, 14.00 (3.50)	15.50 ± 3.25, 15.00 (3.00)	1.44 ± 3.95, 1.00 (4.50)	0.18	**0.04**
Word fluency: Korean	4.86 ± 3.63, 5.00 (5.00)	4.43 ± 5.44, 4.00 (7.00)	−0.43 ± 2.76, 0.00 (3.00)	0.69	9.19 ± 2.86, 8.50 (4.00)	9.81 ± 3.89, 10.50 (5.50)	0.63 ± 4.33, 1.00 (6.00)	0.57	0.49
Stroop test	6.86 ± 4.49, 5.00 (6.00)	7.00 ± 5.72, 6.00 (9.00)	0.14 ± 2.12, 0.00 (4.00)	1.00	14.75 ± 5.59, 17.00 (10.00)	16.75 ± 3.75, 17.50 (6.50)	2.00 ± 5.32, 0.50 (4.50)	0.18	0.44

SNSB-D, Seoul Neuropsychological Screening Battery-Dementia Version; KGT, Kami Guibi-tang, K-BNT; Korean version of Boston Naming Test, SVLT; Seoul Verbal Learning Test. Values are presented as mean ± standard deviation, median (interquartile range). ^†^Based on Wilcoxon signed rank test. ^‡^Based on Wilcoxon rank sum test. *p* values in bold represent significance, *p* < 0.05 was considered statistically significant.

**Table 6:** Changes in SNSB-D subscores between baseline and 24th-weekfollow-up with the aMCI group.

Variable	Mild AD (*n* = 7)				aMCI (*n* = 16)				^‡^ *p* value for treatment difference
	Baseline	24th week	Change	^†^ *p* value	Baseline	24th week	Change	^†^ *p* value	
K-MMSE	22.86 ± 20.29, 22.00 (6.00)	20.29 ± 3.50, 20.00 (7.00)	−2.57 ± 1.51, −2.00 (2.00)	0.03	27.63 ± 1.82, 28.50 (2.50)	27.94 ± 1.65, 28.00 (3.00)	0.31 ± 1.78, 0.50 (1.50)	0.58	**0.01**
SGDS	6.29 ± 3.68, 4.00 (5.00)	5.00 ± 4.58, 4.00 (6.00)	−1.29 ± 2.29, −1.00 (3.00)	0.25	5.75 ± 5.29, 4.00 (9.00)	4.06 ± 4.42, 2.00 (7.00)	−1.69 ± 5.12, −2.00 (4.50)	0.07	0.84
Barthel-ADL	20.00 ± 0.00, 20.00 (0.00)	19.71 ± 0.76, 20.00 (0.00)	−0.29 ± 0.76, 0.00 (0.00)	1.00	20.00 ± 0.00, 20.00 (0.00)	20.00 ± 0.00, 20.00 (0.00)	0.00 ± 0.00, 0.00 (0.00)	—	0.17
K-IADL	0.65 ± 0.39, 0.56 (0.73)	0.86 ± 0.44, 0.80 (0.72)	0.20 ± 0.29, 0.11 (0.61)	0.10	0.13 ± 0.06, 0.10 (0.09)	0.11 ± 0.04, 0.09 (0.01)	−0.02 ± 0.06, 0.00 (0.00)	0.44	**0.02**
CDR-GS	1.00 ± 0.00, 1.00 (0.00)	0.93 ± 0.19, 1.00 (0.00)	−0.07 ± 0.19, 0.00 (0.00)	1.00	0.50 ± 0.00, 0.50 (0.00)	0.34 ± 0.24, 0.50 (0.50)	−0.16 ± 0.24, 0.00 (0.50)	0.06	0.44
CDR-SB	4.86 ± 1.03, 4.50 (1.00)	5.00 ± 1.61, 5.00 (2.50)	0.14 ± 0.69, 0.00 (1.50)	0.53	1.53 ± 0.64, 1.50 (1.00)	1.13 ± 0.62, 1.00 (1.00)	−0.41 ± 0.55, −0.25 (0.50)	**0.01**	0.17
GDS	4.71 ± 0.49, 5.00 (1.00)	4.71 ± 0.49, 5.00 (1.00)	0.00 ± 0.00, 0.00 (0.00)	—	3.00 ± 0.00, 3.00 (0.00)	2.69 ± 0.48, 3.00 (1.00)	−0.31 ± 0.48, 0.00 (1.00)	0.06	0.13

KGT, Kami Guibi-tang; SNSB-D, Seoul Neuropsychological Screening Battery-Dementia Version; K-MMSE, Korean version Mini-Mental State Examination; SGDS, Short Geriatric Depression Scale; Barthel-ADL, Barthel Activities of Daily Living; K-IADL, Korean-Instrumental Activities of Daily Living; CDR-SB, Clinical Dementia Rating Scale–Sum of Boxes; CDR-GS, Clinical Dementia Rating Scale-Global Score; GDS, Global Deterioration Scale. Values are presented as mean ± standard deviation, median (interquartile range). ^†^Based on Wilcoxon signed rank test. ^‡^Based on Wilcoxon rank sum test. *p* values in bold represent significance, *p* < 0.05 was considered statistically significant.

## Data Availability

The datasets used to support the findings of this study are available from the corresponding author upon request.

## Supplementary Materials

Supplementary Materials Table S1: contents of the SNSB-D. Table S2: CONSORT 2010 Checklist.
