# Supplementary material for: Efficacy of Kami Guibi-tang as an Add-On Therapy to Acetylcholinesterase Inhibitor for Cognitive Function in Mild Alzheimer's Disease: A Pilot Study
Source: Evid Based Complement Alternat Med. 2023 Jan 30;2023:4846770. doi: 10.1155/2023/4846770 (PMC9902163; doi:10.1155/2023/4846770)
Supplement: Supplementary Materials — Table S1: contents of the SNSB-D. Table S2: CONSORT 2010 Checklist. [file 4846770.f1.zip › Table S1. Contents of SNSB-D.docx]

**Table S1. Contents of the SNSB-D**

| **Domains / Tests** | **Scores** |
| --- | --- |
| **Attention** | **/ 17** |
| Digit span forward | / 9 |
| Digit span backward | / 8 |
| **Language & Related Function** | **/ 27** |
| K-BNT | / 15 |
| Calculation | / 12 |
| **Visuospatial Function** | **/ 36** |
| Rey copy | / 36 |
| **Memory** | **/ 150** |
| Orientation | / 6 |
| SVLT recall | / 48 |
| SVLT recognition | / 12 |
| Rey recall | / 72 |
| Rey recognition | / 12 |
| **Frontal & Executive Function** | **/ 70** |
| Impersistence | / 3 |
| Contrasting program | / 3 |
| Go-no-go test | / 3 |
| Fist-edge-palm | / 3 |
| Luria loop | / 3 |
| Word fluency: animal | / 20 |
| Word fluency: Korean | / 15 |
| Stroop test | / 20 |
| **Total SNSB-D** | **/ 300** |
